# Overexpression of a Short Sulfonylurea Splice Variant Increases Cardiac Glucose Uptake and Uncouples Mitochondria by Regulating ROMK Activity

**DOI:** 10.3390/life13041015

**Published:** 2023-04-14

**Authors:** Sarah K. El-Meanawy, Holly Dooge, Allison C. Wexler, Anna C. Kosmach, Lara Serban, Elizabeth A. Santos, Francisco J. Alvarado, Timothy A. Hacker, Mohun Ramratnam

**Affiliations:** 1 Division of Cardiovascular Medicine, Department of Medicine, University of Wisconsin School of Medicine and Public Health, Madison, WI 53705, USA; elmeanawy@wisc.edu (S.K.E.-M.);; 2 Cardiology Section, Medical Service, William. S. Middleton Memorial Veterans Hospital, Madison, WI 53705, USA; 3 Cardiovascular Research Center, University of Wisconsin School of Medicine and Public Health, Madison, WI 53705, USA

**Keywords:** K_ATP_ channel, sulfonylurea receptor, renal outer medullary potassium channel, myocardial ischemic reperfusion injury, mitoK_ATP_

## Abstract

The mitochondrial splice variant of the sulfonylurea receptor (SUR2A-55) is associated with protection from myocardial ischemia-reperfusion (IR) injury, increased mitochondrial ATP sensitive K^+^ channel activity (mitoK_ATP_) and altered glucose metabolism. While mitoK_ATP_ channels composed of CCDC51 and ABCB8 exist, the mitochondrial K^+^ pore regulated by SUR2A-55 is unknown. We explored whether SUR2A-55 regulates ROMK to form an alternate mitoK_ATP_. We assessed glucose uptake in mice overexpressing SUR2A-55 (TG^SUR2A−55^) compared with WT mice during IR injury. We then examined the expression level of ROMK and the effect of ROMK modulation on mitochondrial membrane potential (Δψm) in WT and TG^SUR2A−55^ mice. TG^SUR2A−55^ had increased glucose uptake compared to WT mice during IR injury. The expression of ROMK was similar in WT compared to TG^SUR2A−55^ mice. ROMK inhibition hyperpolarized resting cardiomyocyte Δψm from TG^SUR2A−55^ mice but not from WT mice. In addition, TG^SUR2A−55^ and ROMK inhibitor treated WT isolated cardiomyocytes had enhanced mitochondrial uncoupling. ROMK inhibition blocked diazoxide induced Δψm depolarization and prevented preservation of Δψm from FCCP perfusion in WT and to a lesser degree TG^SUR2A−55^ mice. In conclusion, cardio-protection from SUR2A-55 is associated with ROMK regulation, enhanced mitochondrial uncoupling and increased glucose uptake.

## 1. Introduction

Ischemic heart disease is a major burden to society and remains a leading cause of death [1]. One strategy to curtail the progression of ischemic heart disease focuses on reducing cardiac injury during an acute myocardial infarction. While percutaneous interventions to open the occluded infract related artery are effective in reducing disease morbidity, ischemia reperfusion (IR) injury persists. Currently, there is no pharmacologic therapy to treat myocardial IR injury. Major advances in the mechanisms responsible for IR injury point to the mitochondria as a potential therapeutic target [2]. Within the mitochondria, activation of the ATP sensitive mitochondrial K^+^ channel (mitoK_ATP_) prevents IR injury and may be the end effector in multiple pathways [3,4,5]. Thus, deciphering the components and physiologic roles of cardiovascular ATP sensitive K^+^ channel (K_ATP_) subunits will enhance the future discovery of cardio-protective therapies.

Cell surface and inner mitochondrial membranes house K_ATP_ channels that connect the metabolic environment of the cell or organelle to membrane excitability [6,7]. The main components of the ventricular sarcolemmal K_ATP_ (sarcK_ATP_) channel are well accepted and formed by the K^+^ pore Kir6.2, encoded by KCNJ11, and the regulatory subunit SUR2, encoded by ABCC9. The opening of sacrK_ATP_ channels may lead to cardio-protection by stabilizing the resting membrane potential, shortening of the action potential and reducing Ca^2+^ influx [8]. However, many laboratories suggest that mitoK_ATP_ plays a bigger role in cardio-protection than its sarcoplasmic counterpart [4,9]. MitoK_ATP_ is a major component of the mitochondrial K^+^ cycle and contributes to cardio-protection by influencing mitochondrial swelling, and regulating reactive oxygen species generation, ATP production and Ca^2+^ handling [7]. Unfortunately, the existence and components of mitoK_ATP_ are blurrier, with clarification only beginning recently. This makes pharmacologic targeting challenging. The current constituents include coiled-coil domain containing 51 (CCDC51), renal outer medullary K^+^ channel isoform 2 (ROMK2) and the regulatory subunits: ATP binding cassette subfamily B member 8 (ABCB8) and the short splice variant of SUR2A [10,11,12,13].

Recent studies showed that CCDC51 pairs with ABCB8 to form a mitochondrial ATP sensitive K^+^ channel [10]. During this same period, we observed that the overexpression of a short (55 kDa) splice variant of the sulfonylurea receptor isoform 2A (SUR2A-55) led to cardio-protection and enhanced mitoK_ATP_ activity at rest [11]. In addition, we found that SUR2A-55 associates with glucose handling proteins and may confer cardio-protection via glucose metabolism [14]. However, the complete make up of a SUR2A-55-based mitoK_ATP_ channel is incomplete without a pore forming subunit. Thus, pharmacologic targeting remains elusive. Foster et. al. found an isoform of the renal outer medullary K^+^ channel (ROMK) in heart mitochondria [13], which functions as an inwardly rectifying ATP-sensitive K^+^ channel [15,16]. Prior reports suggest that SUR2 isoforms associate with ROMK and regulate its activity [15,17]. While early work demonstrated a role of ROMK in cytoprotection [13], recent work with cardiac specific ROMK knockout mice did not show a role for ROMK in mediating cardio-protection [12]. However, the overexpression of SUR2A-55 may alter ROMKs activity in heart mitochondria, leading to increased K^+^ flux and cardio-protection. Therefore, we hypothesized that cardio-protection observed in mice overexpressing SUR2A-55 may result from increased cardiac mitochondrial ROMK activity and that this altered mitochondrial phenotype may lead to increased glucose uptake. In this investigation, we assessed whether SUR2A-55 affects glucose uptake in myocardial IR injury in mice overexpressing SUR2A-55 (TG^SUR2A−55^). We then evaluated if the activity of sarcK_ATP_ or mitochondrial ROMK channels are altered in TG^SUR2A−55^ mice.

## 2. Materials and Methods

### 2.1. Animal Usage

All animal procedures and experiments were performed with approval from the Institutional Animal Care and Use Committee at the University of Wisconsin. For each experiment, three to six male WT or TG^SUR2A−55^ mice aged 8–12 weeks were used. TG^SUR2A−55^ mice were previously generated by overexpression of a SUR2A-55 construct by the α-myosin heavy chain promoter [11].

### 2.2. Isolated Perfused Hearts

Isolated hearts from mice were perfused according to the Langendorff technique at constant pressure as previously described [11]. Briefly, mice were anesthetized with inhaled 3% isoflurane and then euthanized by cervical dislocation. Hearts were rapidly excised and dissected, and the aorta cannulated with a 22-gauge metal cannula. Hearts were perfused at 37 °C at a constant pressure of 80 mmHg on a custom made Langendorff apparatus with modified Krebs-Henseleit buffer (118 mM NaCl, 4.7 mM KCl, 1.2 mM MgSO_4_, 1.2 mM KH_2_PO_4_, 25 mM NaHCO_3_, 2.5 mM CaCl_2_, 0.5 mM EDTA, 5 mM glucose, and 5 mM sodium pyruvate). Global ischemia was induced by turning off the flow to the isolated mouse heart. Hearts were paced at 360 bpm five minutes into reperfusion to standardize heart rate for glucose uptake experiments.

### 2.3. Cardiac Glucose Uptake

Hearts were subjected to ischemia (45 min) and reperfusion (60 min) and aliquots of the perfusate were taken at 15-, 30-, 45- and 60-min during reperfusion. Glucose uptake was calculated as previously described with the following formula [18].

[Glucose Uptake] (μg/min ∗ mg) = {[G]in − [G]out}

X [Flow Rate] (mL/min)/heart weight (mg)

[G]in: glucose concentration, inflow (μg/mL)

[G]out: glucose concentration, outflow (μg/mL)

### 2.4. Monophasic Action Potential Recordings

Left ventricular monophasic action potentials (MAPs) were recorded from isolated hearts during baseline perfusion and ischemia. MAPs were obtained by using a silver/silver chloride (Ag/AgCl) electrode (Harvard Apparatus, Holliston MA, USA) opposed to the epicardial surface of the free left ventricular wall. Isolated mouse hearts were first secured in a stable position by an opposite side holder (Harvard Apparatus, Holliston MA, USA). The MAP probe was lowered by hand perpendicularly onto the epicardial surface of the heart until gentle but stable contact pressure was achieved. Recordings were accepted for analysis if they satisfied previously documented criteria of MAP amplitude, fast upstroke and stable diastolic baseline [19,20].

### 2.5. Isolation of Mitochondria

Mitochondria from murine ventricular cardiac tissue were isolated by homogenization and differential centrifugation as previously described [21]. In brief, hearts were quickly excised after thoracotomy and placed in ice-cold isolation buffer (50 mM sucrose, 200 mM mannitol, 5 mM KH_2_PO_4_, 1 mM EGTA, and 5 mM 3-(N-morpholino) propanesulfonic acid). Hearts were manually minced and digested for 10 min with 0.1 mg/mL of trypsin, and mitochondria were isolated with differential centrifugation. The final mitochondrial pellet was resuspended in ice cold isolation buffer. For experiments assessing mitochondrial proteins by western blotting, mitochondrial isolates were treated with a protease inhibitor during the isolation process (Pierce Protease Inhibitor, ThermoFisher Scientific, Waltham, MA, USA). Mitochondrial protein concentrations were determined by the Bradford method (Bio-Rad, Hercules, CA, USA).

### 2.6. Western Blot Analysis

Western blots were performed by suspending 30 ug of homogenate tissue in Laemmlii buffer and separating proteins through SDS-PAGE in 4–20% TGX precast gels (Bio-Rad, Hercules, CA, USA). Transfer to PVDF membranes was completed using the iBlot2 Dry Blotting system, after which antibody incubation was carried out in the iBind Flex Western Device (ThermoFisher, Waltham, MA, USA). Membranes were probed with the following primary antibodies: Anti-KCNJ1 (1:500, APC-001, Alomone Labs, Jerusalem, Israel), Anti-CCDC51 (1:1000, HPA010980, Sigma, St. Louis, MO, USA), and Anti-COXIV (1:2000, ab16056, Abcam, Cambridge, UK). The secondary antibody required by all blots was Goat Anti-Rabbit IgG H&L HRP (1:2000, ab205718, Abcam, Cambridge, UK). SuperSignal ECL reagent (Thermo, Waltham, MA, USA) was used to develop membranes which were subsequently imaged with a ChemiDoc MP apparatus (Bio-Rad, Hercules, CA, USA). Band intensity was quantified in ImageLab software (Bio-Rad, Hercules, CA, USA). For quantification, COXIV was used as a loading control.

### 2.7. Mitochondrial Membrane Potential Assessment in Isolated Cardiac Mitochondria

Mitochondrial membrane potential (Δψm) was monitored spectrophotometrically as previously described [21,22,23]. Briefly, using rhodamine 123 (5 nM) and excitation λ_ex_ of 503/510-nm and emission λ_em_ of 527/535-nm for coupled and uncoupled mitochondria respectively, 0.25 mg of mitochondria were added to a cuvette containing malate (5 mM), pyruvate (5 nM) and oligomycin (1 μg/mL). The ATP sensitivity of Δψm was assessed after the addition of 3000 μM of ATP to a cuvette containing no ATP. The ionophore carbonylcyanide-4-trifluoromethoxyphenylhydrazone (FCCP; 10 μM) was used to depolarize the Δψm at the end of each experiment. To assess the effect of ROMK inhibition on resting Δψm and ATP sensitivity, 100 uM of the ROMK inhibitor Compound A (MERCK & Co., Inc., New York, NY, USA) was administered [24]. Rhodamine 123 fluorescence at each time point was normalized to the florescence after the administration of FCCP. Differences between groups were assessed using absolute florescence (a.f.u.) changes after ATP administration.

### 2.8. Isolation of Cardiomyocytes

Mice were dosed with an intraperitoneal injection of 125 Units of Heparin and returned to the carrier for 15 min. Following heparinization, mice were anesthetized with isoflurane and hearts were then rapidly excised, dissected and washed in a 10 cm petri dish containing perfusion buffer (10 mM HEPES, 0.6 mM Na_2_HPO_4_, 113 mM NaCl, 4.7 mM KCl, 12 mM NaHCO_3_, 0.6 mM KH_2_PO_4_, 1.2 mM MgSO_4_-7H_2_O, 10 mM KHCO_3_, 30 mM taurine and 5.5 mM glucose). The aorta was cannulated and secured to a metal canula with 6–0 silk. Hearts were then mounted to a custom built Langendorff apparatus and perfused with perfusion buffer for 5 min at 37 °C at a constant flow rate of 3–4 mL/min. Hearts were then perfused with digestion buffer containing 25 µM CaCl_2_ and 5 mg Liberase for 11–20 min.

Hearts were removed from the cannula and placed in a 30 mm dish containing 4 mL of stopping buffer (18 mL perfusion buffer, 2 mL FBS and 12.5 µM CaCl_2_). The atria were removed and discarded. The ventricles were finely minced and teased apart with fine forceps for two minutes. The solution was pipetted gently with a 2 mm opening pipette, transferred to a 15 mL conical tube through a nylon mesh, and allowed to sediment for 10 min. The supernatant was removed and the pellet was re-suspended in 4 mL stopping buffer. Calcium was reintroduced in increments of 20 µL of 10 mM CaCl_2_, 20 µL of 10 mM CaCl_2_, 40 µL of 10 mM CaCl_2_, 12 µL of 100 mM CaCl_2_ and 20 µL of 100 mM CaCl_2_ for a final concentration of 1 mM. The suspension was mixed well and incubated for 4 min at 37 °C after each addition. The cells were allowed to sediment for 10 min, the supernatant was discarded and the pellet was resuspended in 4 mL of Tyrode’s buffer containing 1 mM CaCl_2_.

### 2.9. Mitochondrial Membrane Potential Assessment in Isolated Cardiomyocytes

Cell suspensions were treated with 4 nM of TMRE, plated on laminin coated, 30 mm glass bottom dishes, and incubated for one hour at room temperature, protected from the light. Plated cardiomyocytes loaded with TMRE were then transferred to a LSM800 confocal microscope (Carl Zeiss, Jena, Germany) in Tyrode’s solution (135 mM NaCl, 4 mM KCl, 1 mM MgCl_2_, 10 mM HEPES, 1.2 mM NaH_2_PO_4_, 10 mM Glucose and 1.8 mM CaCl_2_). Cells were located and positioned in 10× magnification under transmitted light. The plate position was manipulated to find a field with multiple cells. Magnification was increased to 20× and two-dimensional images of TMRE (excitation: 561 nm, emission 565–700 nm) were acquired during the perfusion experiments (Objective: Plan-Apochromat 20×/0.8 M27, interval: 2.53 s, resolution: 512 × 512 px, pixel size: 0.575 µm, pixel time: 4.12 µs, laser intensity: 0.2%, pinhole size 4.75 AU/164 µm, Gain: 550 V). To assess the effect of ROMK inhibition on resting Δψm, TMRE stained isolated cardiomyocytes were perfused with either the ROMK inhibitor, compound A (30 µM), or equivalent vehicle for 4.5 min and continuously recorded. To assess the effect of ROMK inhibition on the response of Δψm to the uncoupler FCCP or the mitoK_ATP_ opener diazoxide, cells were pretreated for 15 min with either 30 µM compound A or equal volume of vehicle, dimethyl sulfoxide (DMSO). Cells were then initially perfused with Tyrode’s solution (0–30 s) and then treated with or without diazoxide (100 µM) for 90 s. Finally, cells were perfused with 10 µM FCCP to uncouple mitochondria until the observation of cell death, marked by cell shrinkage. Total experimental time varied between cells between 15 and 30 min.

### 2.10. Statistical Analysis

Data are reported as mean ± standard error. Statistical analyses were performed using the R program 3.4.2 (R Foundation for Statistical Computing) and Microsoft Excel (Microsoft, Redmond, WA, USA) with a *p* value < 0.05 considered significant. Shapiro–Wilk’s test was used to assess normality. Comparison between two groups was made using the two-tailed Student’s *t*-test or Wilcoxon rank sum test depending on normal distribution of the data sets. Statistical differences between more than two groups were performed by a one-way or two-way ANOVA if assumptions for ANOVA were met.

## 3. Results

### 3.1. TG^SUR2A−55^ Mice Have Increased Glucose Uptake during Reperfusion Compared to WT Mice

**Figure 1:**
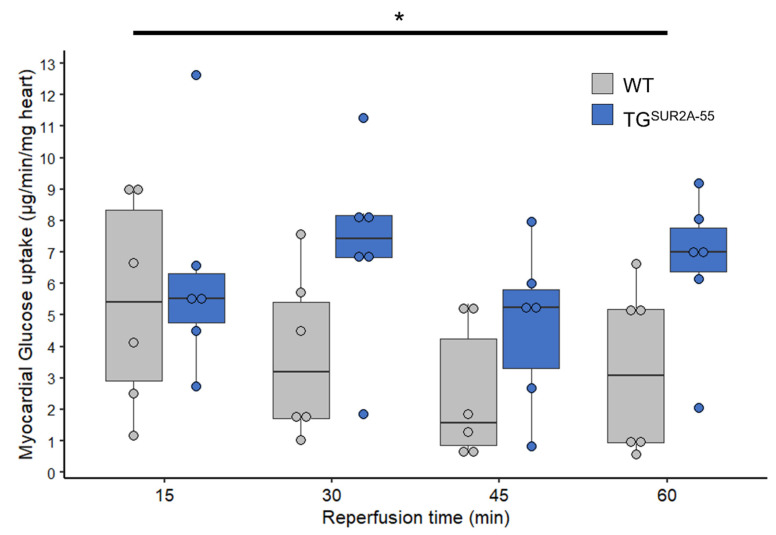
TG^SUR2A−55^ mice have increased glucose uptake during IR injury compared to WT mice. Values represent glucose uptake at time in reperfusion preceded by 45 min of global no f1ow ischemia. In the scatter box plots, boxes cover the 25–75% range of data with the median as a line. *n* = 6/group. * *p* < 0.01. Data were subjected to two-way ANOVA.

We recently observed that TG^SUR2A−55^ mice are protected from IR injury [11] and that SUR2A splice variants associate with cardiac glucose transporter proteins and affect glycolysis [14]. Here, we assessed whether the overexpression of SUR2A-55 can lead to increased glucose uptake during myocardial IR injury. We subjected isolated hearts from TG^SUR2A−55^ and WT littermate controls to ischemia (45 min) and then reperfusion (60 min) and found that TG^SUR2A−55^ mouse hearts had a sustained increase in glucose uptake during reperfusion compared to WT mice (Figure 1, two-way AVONA, *p* < 0.01).

### 3.2. Action Potential Duration during Ischemia in WT and TG^SUR2A−55^ Mice Is Similar

**Figure 2:**
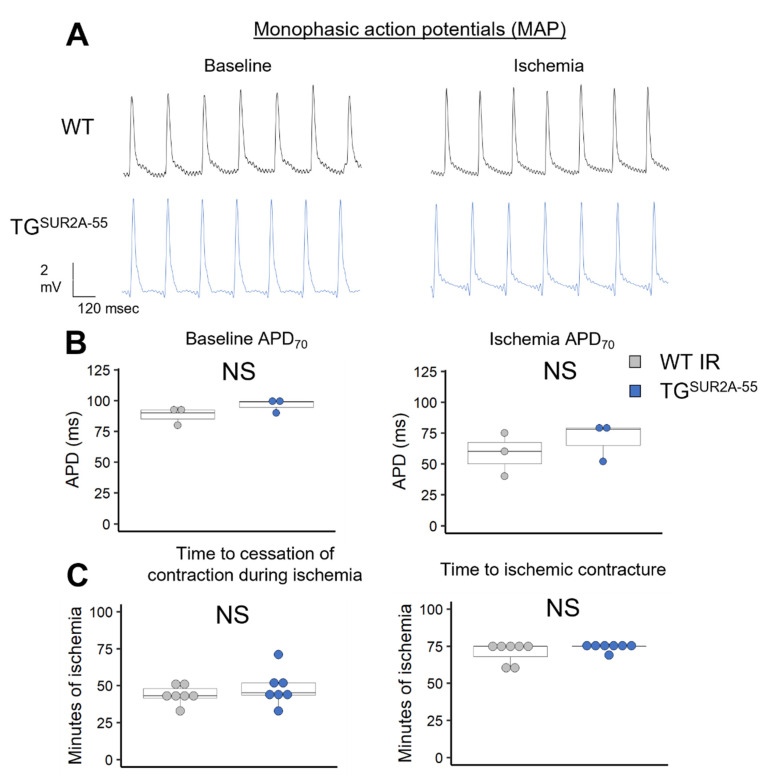
Physiologic assessment of SarcK_ATP_ activity in TG^SUR2A−55^ and WT mice. (**A**) Representative monophasic action potentials from WT and TG^SUR2A−55^ isolated mouse hearts during baseline perfusion and global no flow ischemia. (**B**) Summary data of APD₇₀ showing no change between WT and TG^SUR2A−55^ mice during baseline perfusion and ischemia. (**C**) There was no change in the time to cessation of contraction and ischemic contracture after the start of ischemia between WT and TG^SUR2A−55^ mice. In the scatter box plots, boxes cover the 25–75% range of data with the median as a line. *n* = 3/group for MAP and *n* = 6/group for contracture experiments. NS = not significant.

Prior studies reported that the SUR2A-55 splice variant can assemble with Kir6.1 and Kir6.2 to form functional K_ATP_ channels [25]. SUR2A-55-based channels are more resistant to nucleotide inhibition and pharmacologic modulation than channels formed by the full length SUR2A protein. While the SUR2A-55 splice variant contains a mitochondrial targeting signal, the possibility remains that significant overexpression in mouse hearts may form sarcK_ATP_ channels that are more active and as a result mediate cardio-protection and glucose metabolism [11]. To investigate the physiologic function of sarcK_ATP_ channels in TG^SUR2A−55^ compared with WT mice, we assessed action potential duration, time to cessation of contraction and time to ischemic contracture in isolated hearts subjected to ischemia (Figure 2). Increased activation of cardiac sarcK_ATP_ channels leads to action potential shortening, reduced contractility and may lead to cardio-protection from energy conservation [3,8]. The action potential duration was similar in WT and TG^SUR2A−55^ mice at baseline (94.8 ± 5 vs. 87.4 ± 10 ms, NS) and after ischemia (67.5 ± 10 vs. 57.5 ± 18 ms, NS). There was also similar time to cessation of contraction (43.7 ± 3 vs. 48.6 ± 4 min, NS) and time to ischemic contracture (70.9 ± 3 vs. 74.3 ± 3 min, NS) in both mice genotypes, suggesting no physiological difference in sarcK_ATP_ activity during ischemia.

### 3.3. Mitochondrial Protein Expression of mitoK_ATP_ K^+^ Channel Pore Candidates in WT and TG^SUR2A−55^ Mice

**Figure 3:**
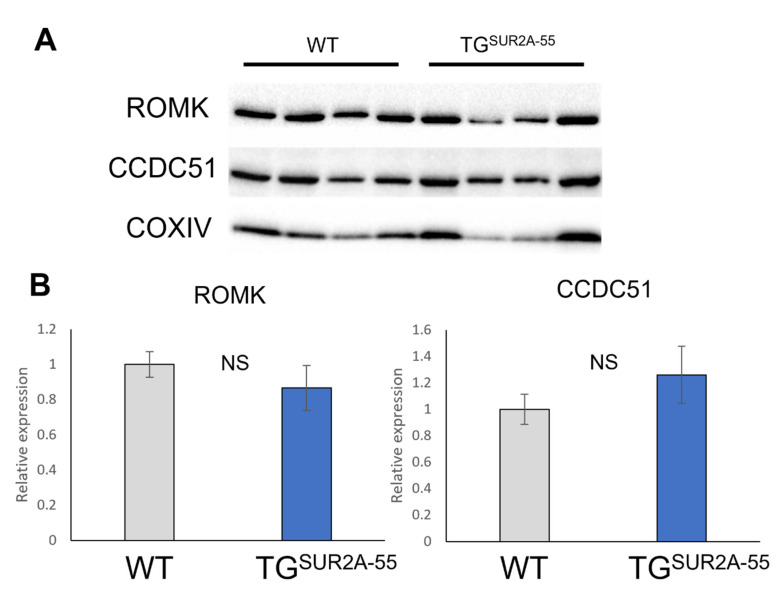
Assessment of mitoK_ATP_ potassium pore expression. (**A**) Representative images of western blots. (**B**) Quantification of bands illustrates similar cardiac mitochondrial protein expression between WT and TG^SUR2A−55^ of ROMK and CCDC51. Data normalized to COXIV expression. Data presented as the means + SEMs. *n* = 4/group NS = not significant.

We did not identify a significant effect from the overexpression of SUR2A-55 on sarcK_ATP_ channel activity and, therefore, examined the protein expression of candidate mitoK_ATP_ channels in TG^SUR2A−55^ compared to WT mice (Figure 3). Functional K_ATP_ channels only occur if both K^+^ channel pore and regulatory subunits are expressed [6]. In order to increase functional mitoK_ATP_ channels, we hypothesize that the overexpression of SUR2A-55 may lead to an increase in detectable mitoK_ATP_ channel pores. We found that the known mitoK_ATP_ K^+^ channel pores, ROMK and CCDC51, are present in WT and TG^SUR2A−55^ mice. However, we did not observe significant differences in ROMK or CCDC51 protein expression between WT and TG^SUR2A−55^ mouse heart mitochondria. (ROMK, 1 ± 0.072 vs. 0.866 ± 0.128, respectively; NS, and CCDC51 1 ± 0.114 vs. 1.26 ± 0.217, respectively; NS).

### 3.4. Effect of ROMK Inhibition on Δψm in Isolated Cardiomyocytes from WT and TG^SUR2A−55^ Mice

**Figure 4:**
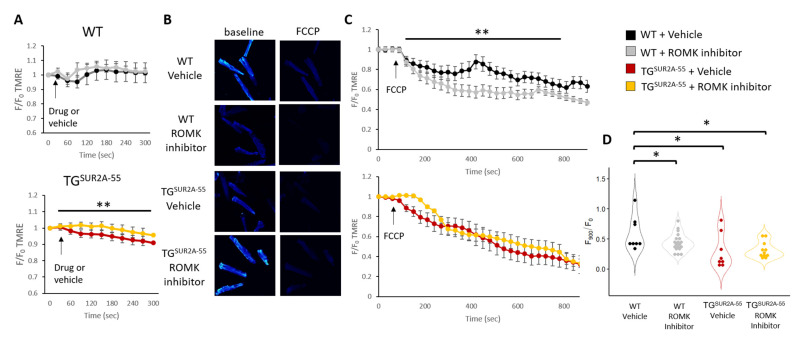
Δψm in isolated cardiomyocytes from WT and TGSUR2A-55 mice treated with or without a ROMK inhibitor (**A**) Δψm during ROMK inhibitor or vehicle perfusion. (**B**) Confocal images of vehicle or ROMK inhibitor treated isolated adult mouse cardiomyocytes stained with TMRE at baseline and after FCCP perfusion at 20× magnification. (**C**) Cumulative data of TMRE fluorescence relative to baseline (F/F_o_) of vehicle or ROMK inhibitor treated cardiomyocytes during 10 μM FCCP perfusion. (**D**) Summary data from (**C**) at end FCCP perfusion. Only cells determined to be alive by morphology (not shrunken) were assessed at end FCCP perfusion. Data presented as the means ± SEMs. For panel (**A**), *n* = 8–10 cells/group; 3 mice/group; for panel (**C**), *n* = 11–33 cells/group; 4 mice/group. * *p* < 0.05; ** *p* < 0.01. Data were subjected to two-way ANOVA (**A**,**C**) or Student’s *t*-test (**D**).

Prior studies have suggested that mice overexpressing the SUR2A-55 splice variant have a protective mitochondrial phenotype and a more active mitoK_ATP_ channel [21]. TG^SUR2A−55^ mouse heart Δψm are more depolarized at rest and are less sensitive to K_ATP_ pharmacology [11]. These findings suggest heightened mitoK_ATP_ activity in this mouse model. Thus, the TG^SUR2A−55^ mouse represents an interesting model to test pharmacologic manipulators of mitoK_ATP_. While there was no significant change in protein expression of ROMK, SUR2 splice variants are known to regulate this channel in the kidney. Therefore, we continued to investigate whether SUR2A-55 may regulate ROMK activity by assessing Δψm in cardiomyocytes. First, we examined the effect of ROMK inhibitor treatment on resting Δψm in isolated cardiomyocytes from WT and TG^SUR2A−55^ mice. We found no significant change in the Δψm from WT isolated cardiomyocytes during ROMK inhibitor perfusion. However, we found that Δψm from TG^SUR2A−55^ isolated cardiomyocytes hyperpolarize after ROMK inhibition compared to vehicle (Figure 4A).

Interestingly, while we did not observe a significant effect on baseline Δψm in WT isolated cardiomyocytes after ROMK inhibition, we found that ROMK blockade depolarized Δψm to a greater extent than vehicle treated cardiomyocytes in WT mice after perfusion with the uncoupler FCCP (Figure 4C). We also found that TG^SUR2A−55^ isolated cardiomyocyte Δψm depolarized to a greater extent than WT control mice (Figure 4D), but that ROMK inhibition had no additional effect in TG^SUR2A−55^ after FCCP treatment (Figure 4C,D). Our results suggest that ROMK channels, while closed at rest in WT mice, are open in TG^SUR2A−55^ mice. ROMK channels also appear to mediate mitochondrial uncoupling in WT mice, but may switch to a more closed state in TG^SUR2A−55^ mice during mitochondrial stress, leading to increased mitochondrial uncoupling. The uncoupling of mitochondria is associated with cardio-protection likely from a reduction in reactive oxygen species [26,27,28] and, thus, may represent a mechanism for protection in TG^SUR2A−55^ mice after IR injury.

### 3.5. Effect of ROMK Inhibition after Diazoxide Treatment on Δψm in Isolated Cardiomyocytes from WT and TG^SUR2A−55^ Mice

**Figure 5:**
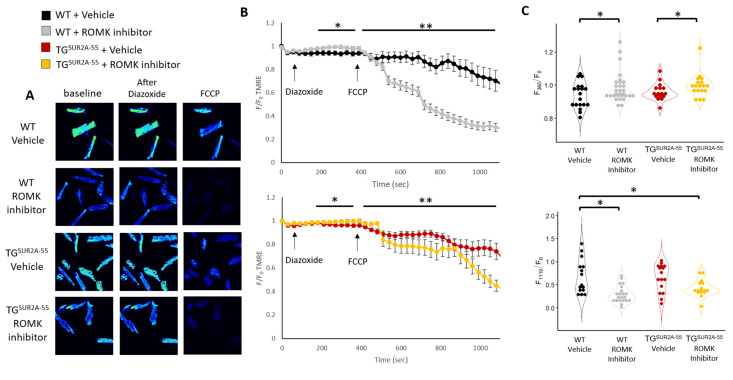
The influence of ROMK inhibition on Δψm in isolated adult mouse cardiomyocytes from WT and TG^SUR2A−55^ mice treated with diazoxide. (**A**) Confocal images of vehicle or ROMK inhibitor treated cardiomyocytes stained with TMRE at baseline, after diazoxide (100 μM) perfusion, and FCCP (10 μM) perfusion at 20× magnification. (**B**) Cumulative data of TMRE fluorescence relative to baseline (F/Fe) of vehicle or ROMK inhibitor treated cardiomyocytes after diazoxide followed by FCCP perfusion. (**C**) Summary data of TMRE florescence after diazoxide (top) and FCCP perfusion (bottom). Data presented as the means ± SEMs. *n* = 16–30 cells/group; 4 mice/group. * *p* < 0.05; ** *p* < 0.01. Data were subjected to two-way ANOVA (**B**) or Student’s I-test (**C**).

To further assess whether SUR2A-55 regulates ROMK activity, the mitoK_ATP_ opener diazoxide was used to activate channels. Diazoxide has been shown in prior reports to stimulate ROMK activity [13,16]. Isolated cardiomyocytes from WT and TG^SUR2A−55^ mice treated with or without the ROMK inhibitor were perfused with diazoxide to first investigate difference in basal Δψm (Figure 5). We found that ROMK blockade prevents a mild diazoxide induced depolarization of the Δψm in both WT and TG^SUR2A−55^ cardiomyocytes. (WT; Vehicle treated 6.2% ± 1.8 vs. ROMK inhibitor treated 1.8% ± 1.6 change in florescence normalized to baseline (F/F0), *p* = 0.04, TG^SUR2A−55^; Vehicle treated 4.5% ± 1.3 vs. ROMK inhibitor treated 0.5% ± 0.2 change in florescence normalized to baseline (F/F0), *p* = 0.04). We also found that ROMK inhibitor treated WT isolated cardiomyocytes had pronounced uncoupling with FCCP compared to vehicle treated cells after diazoxide perfusion. Diazoxide has been shown to preserve Δψm [29] and our results suggest this occurs via a ROMK dependent fashion. While TG^SUR2A−55^ heart mitochondria treated with the ROMK inhibitor demonstrated an increase in Δψm depolarization compared to vehicle treated, it is blunted compared to WT mice. Our prior results suggest SUR2A-55-based channels have reduced diazoxide sensitivity and, therefore, may explain the reason for smaller differences between Δψm from TGSUR2A-55 cardiomyocyte treated with vehicle and ROMK inhibition.

### 3.6. Effect of ROMK Inhibition on mitoK_ATP_ Activity in Isolated Heart Mitochondria from WT and TG^SUR2A−55^ Mice

**Figure 6:**
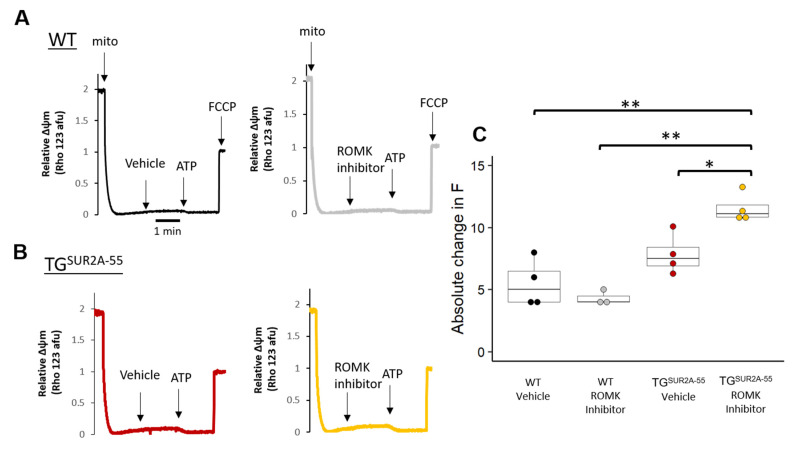
The effect of ROMK inhibition on Δψm in WT and TG^SUR2A−55^ mice isolated cardiac mitochondria. (**A**,**B**) show representative experiments of Δψm measured by rhodamine 123 fluorescence in isolated mitochondria from WT and TG^SUR2A−55^ mouse hearts treated with vehicle or ROMK inhibitor and then 3000 μM ATP. Graphs are normalized to florescence after the addition of FCCP. (**C**) Summary data of Δψm after ROMK inhibitor treatment vs. vehicle and response to ATP. In the scatter box plots, boxes cover the 25–75% range of data with the median as a line. *n* = 4 mice/group, * *p* < 0.05, ** *p* < 0.001.

Our results suggest that the effects of diazoxide are blocked by ROMK inhibition. We then assessed whether the ROMK inhibitor could alter Δψm from isolated heart mitochondria. We tested to see if ROMK inhibition was able to hyperpolarize the resting Δψm of isolated mitochondria in the presence of no ATP. We also assessed whether ROMK inhibition was able to block the effect of Δψm hyperpolarization with the addition of ATP due to closure of the mitoK_ATP_ channel. In the presence of no ATP, ROMK inhibition had no significant effect on cardiac Δψm from WT or TG^SUR2A−55^ mice (Figure 6). While there was no change in the ATP sensitivity of WT Δψm due to ROMK inhibition, we paradoxically found that ROMK inhibition increased the ATP sensitivity of Δψm in TG^SUR2A−55^ mouse hearts (7.8 ± 1 vs. 11.6 ± 1 a.f.u., *p* = 0.005). These results suggest that ROMK may not be the classical mitoK_ATP_ channel, but may regulate mitochondrial K+ channels such as CCDC51 in the setting of SUR2A-55 overexpression.

## 4. Discussion

We found that TG^SUR2A−55^ mice have increased glucose uptake compared with WT control mice during myocardial IR injury. We also found that TG^SUR2A−55^ mice have increased basal ROMK activity which is not observed in WT mice. Interestingly, we found that SUR2A-55 overexpression and ROMK blockade in WT mice enhance mitochondrial uncoupling. In addition, we found that ROMK inhibition prevents diazoxide mediated Δψm depolarization in WT isolated resting cardiomyocyte and that preservation of Δψm by diazoxide is abolished by ROMK blockade. The above effects are again muted in the TG^SUR2A−55^ mouse line. However, in isolated mitochondria from both WT and TG^SUR2A−55^ mice, ROMK does not reduce ATP sensitivity, paradoxically increasing it in the TG^SUR2A−55^ mouse line. Our results indicate that SUR2A-55 overexpression regulates ROMK activity, promotes uncoupled mitochondria and modulates glucose metabolism during cardiovascular stress. By reducing mitochondrial ATP production from uncoupling mitochondria, there is a greater reliance on cytoplasmic glycolytic ATP synthesis. Unexpectedly, our results also suggest a role for cardiac ROMK in mitochondrial coupling. It appears that TG^SUR2A−55^ mice have increased ROMK channel activity at baseline which closes during mitochondrial stress leading to a more uncoupled state. In conclusion, we propose that SUR2A-55 participates in ROMK regulation leading to uncoupling of mitochondrial and switching metabolism to cytoplasmic ATP production.

K_ATP_ channels are ubiquitously found in human tissues [30,31,32,33]. SarcK_ATP_ channels were first described in cardiomyocytes [34] and found to connect the intracellular metabolic state with membrane excitability. Opening sacrK_ATP_ channels increases K^+^ flux through Kir6.x pore subunits. The increase in K^+^ entry stabilizes the resting membrane potential, shortens the action potential and reduces contractility [8]. In mitochondria, K_ATP_ channels regulate mitochondrial volume which, in turn, can modulate respiration and ROS production [7]. K_ATP_ channels are hetero-octameric protein complexes composed of four pore forming inward rectifying K^+^ channel subunits and four regulatory subunits. Their biophysical properties are determined by their unique subunits and reflect the different functional needs of each tissue and organelle. Splice variants of the regulatory subunit SUR are noted in a number of reports [35]. The SUR2A-55 splice variant targets mitochondria, leads to a heightened mitoK_ATP_ channel, and promotes cardio-protection [11,36]. In addition, we recently found that SUR2A-55 can associate with GLUT transporters [14] and in this report found that SUR2A-55, when overexpressed in mouse hearts, increases glucose uptake during IR injury. The interaction between K_ATP_ channels and glucose metabolism, specifically glycolysis, has been reported in multiple laboratories [6]. ATP produced by glycolysis preferentially regulates channel activity [37]. Proteomic work along with two-hybrid and coimmunoprecipitation assays demonstrated an interaction between glycolytic enzymes and K_ATP_ channels [6]. Our work supports a role for SUR2A-55 in glucose metabolism as well. The reasons for cardio-protection from SUR2A-55 overexpression may involve both mitochondrial K_ATP_ modulation and cardiomyocyte glucose metabolism.

While effects on glucose metabolism from SUR2A splice variants exist, this effect may be downstream from K_ATP_ channel activation. In this report, we assessed whether the SUR2A-55 splice variant affects ventricular K_ATP_ channels. We did not observe significant changes in sarcK_ATP_ activity or the expression levels of mitoK_ATP_ K^+^ pore subunits. We targeted the ROMK channel in our physiological experiments, as prior reports in the kidney show an association with SUR isoforms [17]. In addition, protein binding partners for ROMK are unknown in mitochondria but have been identified for CCDC51 [10]. Our work suggests that SUR2A-55 can regulate ROMK and that ROMK is important in mitochondrial coupling. The blockade of ROMK in WT cardiomyocytes depolarizes Δψm to a greater extent when mitochondria are stressed with the protonophore FCCP. This observation is also present and appears heightened in the setting of diazoxide. The TG^SUR2A−55^ mouse line also has increased uncoupling of mitochondria compared to WT mice. ROMK blockade does not increase this effect, suggesting that ROMK channel activity is already reduced in challenged TG^SUR2A−55^ mice heart mitochondria. Prior studies have reported that mild uncoupling of mitochondria leads to cardio-protection with a reduction in reactive oxygen species generation [26,28]. We postulate that reduced ROMK activity in stressed TG^SUR2A−55^ mice mitochondria leads to enhanced uncoupling. With the enhanced uncoupling of mitochondria, ATP production shifts to the cytosol. Therefore, a compensatory increase in ATP production from glycolysis may lead to greater glucose utilization. While more studies will be needed to support this mechanism, our current report offers a reason for SUR2A-55 induced cardio-protection. It is also possible that SUR2A-55 and other SUR proteins do not rely on their K^+^ pairing partner to regulate glucose metabolism. Thus, a non-K_ATP_ channel mechanism for SUR2 may exist that independently regulates glucose metabolism in cardiomyocytes.

Currently, the reason for the existence of ROMK in heart mitochondria is unclear. Early results showed a role of ROMK in cytoprotection [13], but more recent results do not support a role for ROMK in cardio-protection [12]. We did find a diazoxide sensitive component to ROMK inhibition similar to others [13,16]. Interestingly, we found that Δψm depolarizes more rapidly in ROMK-treated WT mice than vehicle treated mice when given the H+ ionophore, FCCP. While this may be related to interactions between the ROMK inhibitor and FCCP, recent studies have shown that FCCP interacts with the ADP/ATP carrier (AAC) to regulate H+ transport in mitochondria [38]. Therefore, a physiologic connection between ROMK and AAC may exist. In addition, compensatory mechanisms to preserve Δψm may be unavailable by ROMK inhibition. The function of ATP synthase in reverse mode is one such mechanism that preserves the H^+^ gradient, though at the cost of ATP consumption [39]. In our isolated mitochondrial preparations, we blocked ATP synthase by oligomycin to isolate mitoK_ATP_ activity, but we did not block ATP synthase in our isolated cardiomyocyte perfusion experiments. Prior work has shown that ROMK co-localizes with ATP synthase [13]. Further work to see if ROMK associates with ACC or complex V of the electron transport chain may be a reasonable avenue to pursue.

While our study demonstrates the effect of SUR2A-55 overexpression on K_ATP_ channel activity and glucose uptake, there are limitations. First, we assessed glucose uptake in an ex vivo isolated heart model instead of an in vivo model. Thus, translating our findings to whole organisms undergoing cardiac stress may be slightly limited. Second, the assessment of mitoK_ATP_ activity with Δψm may not be as sensitive as other assays, such as mitochondrial swelling or thallium flux experiments. Third, it is possible that SUR2A-55 associates with other unknown mitochondrial K_ATP_ channels or CCDC51. Downstream signaling of an unidentified SUR2A-55 channel complex may lead to increased glucose uptake. We used physiologic responses to test interactions between SUR2A-55 and sarcK_ATP_ and ROMK. While a physical connection may exist as well, we believed a physiologic connection would be more relevant. Finally, we used a pharmacologic approach in our mouse model experiments rather than genetic means to induce a loss of function ROMK state. This may lead to off-target effects. In addition, we used non-diseased mouse models. Rodent models of diabetes (i.e., female ZDF-1 rate) may be a better model to assess the impact of cardiometabolic derangements.

In humans, loss of function mutations in ABCC9, the gene responsible for SUR2, are associated with the development of dilated cardiomyopathy [40], atrial fibrillation [41] and ABCC8-related Intellectual disability Myopathy Syndrome (AIMS) [42]. While the gain and loss of function mutations in Kir6.2 have been well studied and reported, there is no significant evidence for cardiac abnormalities in these patients [43]. In addition, humans with ROMK loss of function mutations that lead to kidney disease and the Barters syndrome [15] do not appear to have a significant cardiac phenotype. Mice with Kir6.2 deletion have a similar basal cardiac phenotype compared to WT but fair worse during cardiovascular stress [3,44,45]. SUR2 gene mutations in mice have a varied phenotype depending on the genetic strategy undertaken. Mice with genetic disruption of exons 12–16 encoding the first nucleotide binding domain of SUR2 are protected from myocardial IR injury [46]. However, mice with exon 5 disruption, which leads to both deletion of plasma membrane and mitochondrial inner membrane SUR2A isoforms, develop a severe neonatal cardiomyopathy with mitochondrial immaturity [47]. These human genetic and mouse model observations along with our results support the idea that SUR2, and, namely, SUR2A-55, participates in a complex network of K_ATP_ channel regulation. Future research aimed at clarifying the role of sulfonylurea receptor modulation on metabolism will greatly enlighten our understanding of cardiometabolic disease.

## 5. Conclusions

In conclusion, the ventricular sulfonylurea receptor is a unique component of K_ATP_ channels with different physiologic effects determined by various splice variants. Our work alongside prior reports suggest a link between the sulfonylurea receptor and glucose and mitochondrial metabolism. The cardiac SUR2A-55 splice variant promotes cardiac protection from ischemia reperfusion injury by uncoupling mitochondria and leading to enhanced glucose uptake. This is partly accomplished by blocking ROMK channels which are important in mitochondrial coupling during stress. The connection that links mitochondrial sulfonylurea receptor proteins, ROMK activity and mitochondrial uncoupling with glucose metabolism is an important mechanism in cardiac protection. Further research to elucidate the pathways that link mitochondrial and cytoplasmic glucose metabolism by cardiac sulfonylurea receptors may lead to novel therapies in heart disease.

## Data Availability

Data available on request to the corresponding author.
